# *LOXL1* variants in elderly Japanese patients with exfoliation syndrome/glaucoma, primary open-angle glaucoma, normal tension glaucoma, and cataract

**Published:** 2008-10-27

**Authors:** Masaki Tanito, Masayoshi Minami, Masakazu Akahori, Sachiko Kaidzu, Yasuyuki Takai, Akihiro Ohira, Takeshi Iwata

**Affiliations:** 1 Department of Ophthalmology, Shimane University Faculty of Medicine, Izumo, Shimane, Japan; 2 National Institute of Sensory Organs, National Hospital Organization Tokyo Medical Center, Tokyo, Japan

## Abstract

**Purpose:**

To evaluate the association of lysyl oxidase like 1 (*LOXL1*) gene variants in Japanese patients with open-angle glaucoma.

**Methods:**

We evaluated the association of three *LOXL1* variants (rs1048661, rs3825942, and rs2165241) in 142 Japanese patients with exfoliation syndrome (EX; n=59) and exfoliation glaucoma (EG; n=83) as well as in 251 control patients aged 70 years or older with primary open-angle glaucoma (PG; n=40), normal tension glaucoma (NG; n=54), and cataract (CT; n=157).

**Results:**

In comparison with the CT group, the single nucleotide polymorphisms (SNPs) showed significant association with EX, EG, and EX+EG. The odds ratio (OR)=19.71–28.23 and p=1.69x10^−23^-3.00x10^−45^ for allele T of rs1048661; OR=28.21–39.78 and p=1.77x10^−8^-2.42x10^−22^ for allele G of rs3825942; and OR=16.59–23.40 and p=4.79x10^−5^-1.08x10^−9^ for allele C of rs2165241. In comparison with the controls (CT+PG+NG), the haplotype rs1048661/rs3825942 (T/G) was significantly associated with EX+EG (p=8.27x10^−44^), and haplotype G/A had a significant protective effect (p=2.25x10^−14^). None of the three SNPs showed significant differences between the EX and EG groups or between the PG and NG groups.

**Conclusions:**

These SNPs are associated with exfoliation syndrome/glaucoma in the Japanese population. The risk alleles in rs1048661 and rs2165241 are different from other populations. Additional genetic or environmental risk factors other than these *LOXL1* SNPs could be associated with the development of exfoliation syndrome as well as exfoliation glaucoma among exfoliation syndrome patients.

## Introduction

Exfoliation syndrome is the most common identifiable cause of open-angle glaucoma worldwide. It is an age-related, generalized disorder of the extracellular matrix that is characterized by the production and progressive accumulation of a fibrillar extracellular material in many ocular tissues [1]. A recent genome-wide association study demonstrated that one intronic single nucleotide polymorphism (SNP, rs2165241) and two exonic SNPs (rs1048661 [R141L] and rs3825942 [G153D]), which are located in the first exon of the lysyl oxidase like1 (*LOXL1*) gene on chromosome 15q24.1, are highly associated with exfoliation syndrome in Icelandic and Swedish populations while none of these SNPs were associated with primary open-angle glaucoma in either of the two populations [2].

Until now, several lines of studies have reported significant associations of these *LOXL1* SNPs with exfoliation syndrome [3-16] or the lack of associations of these SNPs with primary open-angle glaucoma [16-18] in various populations. In these studies, subjects in their 40s and 50s are sometimes recruited as controls. The prevalence of clinical exfoliation syndrome increases with age, particularly after the age of 60 years [1]. However, preclinical exfoliation syndromes in younger generations are able to distinguish from normal subjects until patients become older. Accordingly, the level of statistical significance between exfoliation syndrome and controls could be underestimated in studies that include younger patients in the control group. In this study, we evaluated the association of three *LOXL1* SNPs in Japanese patients with exfoliation syndrome/glaucoma, primary open angle glaucoma, normal tension glaucoma, and cataract. To reduce the chance of misclassifying latent syndromes within the control group, only patients aged 70 years or older were recruited as primary open-angle glaucoma, normal tension glaucoma, or cataract subjects in this study. To the best of our knowledge, this is the first study reporting an association of *LOXL1* SNPs with exfoliation syndrome/glaucoma using age-controlled subjects. This is also the first study reporting the association of *LOXL1* SNPs with normal tension glaucoma.

## Methods

### Subjects

Three hundred and ninety-three unrelated Japanese subjects presenting exfoliation syndrome without glaucoma (EX), exfoliation syndrome with glaucoma (EG), primary open-angle glaucoma (PG), normal tension glaucoma (NG), and cataract (CT) were recruited at the Shimane University Hospital and Iinan Hospital in Shimane, Japan. This study adhered to the tenets of the Declaration of Helsinki. The research was reviewed and approved by the Institutional Review Boards of both hospitals. Written informed consent was obtained from all of the subjects. All of the subjects underwent a dilated pupil examination of the anterior segments, ocular media, and fundus using a slit-lamp and a funduscope. The subjects with EX and EG exhibited the typical pattern of exfoliation material on the anterior lens surface and/or pupillary margin during slit-lamp examination. The subjects with EG and PG had a history of intraocular pressure (IOP) greater than or equal to 21 mmHg, and the subjects with EX, NG, and CT had a history of IOP never exceeding 20 mmHg. The subjects with PG, NG, and EG presented a typical glaucomatous optic disc cupping or rim thinning and visual field loss. The patients who had a history of IOP greater than or equal to 21 mmHg but no glaucomatous optic disc changes nor visual field loss were assigned as EG in this study. To avoid possible misclassification of latent exfoliation syndromes as PG, NG, or CT, patients younger than 70 years old were not recruited. The number of subjects, each gender, and the mean and range of ages in each group are summarized in Table 1.

**Table 1:** Summary of study populations.

**Parameter**	**Exfoliation syndrome or glaucoma**			**Primary glaucoma**			**CT**	**p value**
	**EX**	**EG**	**EX+EG**	**PG**	**NG**	**PG+NG**		
No. of subjects	59	83	142	40	54	94	157	
Male:Female								
N	12:47	42:41	54:88	14:26	18:36	32:62	45:112	0.2264*
%	20.3:79.7	50.6:49:4	50.6:49:4	35.0:65.0	33.3:66.7	34.0:66.0	28.7:71.3	
Age (years)								
mean±SD	78.2±8.0	78.8±8.5	78.5±8.2	75.6±5.3	78.3±4.8	77.2±5.1	77.2±5.0	0.1271**
Range	55–95	57–95	55–95	70–87	70–91	70–91	70–90	

The asterisk and double asterisk indicate that the p values were obtained by Pearson's χ^2^test and one-way ANOVA, respectively, among three (EX+EG, PG+NG, and CT) groups. EX, exfoliation syndrome; EG, exfoliation glaucoma; PG, primary open-angle glaucoma aged 70 years or older; NG, normal tension glaucoma aged 70 years or older; CT, cataract aged 70 years or older.

### DNA genotyping

Genomic DNA was extracted from the peripheral white blood cells of each subject. Polymerase chain reaction (PCR) was performed using primers designed to amplify the genomic region containing both rs1048661 and rs3825942 (forward primer: 5′-AGG TGT ACA GCT TGC TCA ACT C-3′ and reverse primer: 5′-TAG TAC ACG AAA CCC TGG TCG T-3′) or just rs2165241 (forward primer: 5′-AGA ATG CAA GAC CTC AGC ATG AG-3′ and reverse primer: 5′-TAG TGG CCA GAG GTC TGC TAA G-3′). The sequence was determined based on the dideoxy terminator method using an ABI PRISM 3130xl Genetic Analyzer (Applied Biosystems, Foster City, CA) according to the manufacturer’s protocol. We used SeqScape Software version 2.5 (Applied Biosystems) to analyze the sequence alignment.

### Statistical analysis

The deviation of the genotype distributions from the Hardy–Weinberg equilibrium was assessed in the case and control samples using HAPLOVIEW version 4.0 [19]. Statistical analysis was performed using R version 2.6.2. Fisher’s exact test was used to compare the allele or genotype frequencies of each case group with the controls. The odds ratio (OR) and 95% confidence intervals (CIs) were calculated by the logistic regression method. Individual haplotypes and their estimated population frequencies were inferred using HAPLOVIEW version 4.0 [19] with all of the parameters set at the default values.

## Results

The allelic and genotypic counts and frequencies of SNPs rs1048661, rs3825942, and rs2165241 within *LOXL1* are shown in Table 2. The ORs and p values for the allelic and genotypic frequencies of the three SNPs in comparison between the cases (EX, EG, and EX+EG) and controls (CT, PG, NG, and PG+NG) are shown in Table 3. In comparison with the CT group, the SNPs showed a significant association with EX, EG, and EX+EG for the T allele of rs1048661 at OR=19.71–28.23 and p=1.69x10^−23^-3.00x10^−45^, the G allele of rs3825942 at OR=28.21–39.78 and p=1.77x10^−8^-2.42x10^−22^, and the C allele of rs2165241 at OR=16.59–23.40 and p=4.79x10^−5^-1.08x10^−9^ (Table 3). The genotypes, TT of rs1048661 (p=4.11x10^−25^-3.78x10^−43^), GG of rs3825942 (p=3.53x10^−11^-2.10x10^−33^), and CC of rs2165241 (p=1.95x10^−4^-1.07x10^−8^), also showed significant associations with EX, EG, and EX+EG when they were compared to the CT group (Table 2 and Table 3). Significant associations with EX, EG, and EX+EG were detected in comparisons with the primary glaucoma groups (PG, NG, and PG+NG) for the alleles, T of rs1048661, G of rs3825942, and C of rs2165241, as well as the genotypes, TT of rs1048661, GG of rs3825942, and CC of rs2165241, with the exception of the comparisons between EX, EG, or EX+EG and the PG groups for allelic (OR=4.56–6.43 and p=0.3051–0.0729) and genotypic (p=0.3000–0.0715) frequencies in rs2165241 (Table 3).

**Table 2:** Allelic and genotypic counts and frequencies of rs1048661, rs3825942, and rs2165241 in exfoliation syndrome/glaucoma (EX, EG, and EX+EG), primary glaucoma (PG, NG, PG+NG), and cataract (CT).

** **	**Exfoliation syndrome or glaucoma**						**Primary glaucoma**							
	**EX**		**EG**		**EX+EG**		**PG**		**NG**		**PG+NG**		**CT**	
	**Count**	**Frequency**	**Count**	**Frequency**	**Count**	**Frequency**	**Count**	**Frequency**	**Count**	**Frequency**	**Count**	**Frequency**	**Count**	**Frequency**
rs1048661														
Allele														
T	111	0.941	159	0.958	270	0.951	39	0.488	50	0.463	89	0.473	140	0.446
G	7	0.059	7	0.042	14	0.049	41	0.513	58	0.537	99	0.527	174	0.554
Genotype														
TT	54	0.915	76	0.916	130	0.916	10	0.25	8	0.148	18	0.192	25	0.159
TG	3	0.051	7	0.084	10	0.070	19	0.475	34	0.630	53	0.564	90	0.573
GG	2	0.034	0	0	2	0.014	11	0.275	12	0.222	23	0.245	42	0.268
rs3825942														
Allele														
G	117	0.992	165	0.994	282	0.993	64	0.800	86	0.796	150	0.798	253	0.806
A	1	0.009	1	0.006	2	0.007	16	0.200	22	0.204	38	0.202	61	0.194
Genotype														
GG	58	0.983	82	0.988	140	0.986	25	0.625	33	0.611	58	0.617	100	0.637
AG	1	0.017	1	0.012	2	0.014	14	0.350	20	0.370	34	0.362	53	0.338
AA	0	0	0	0	0	0	1	0.025	1	0.019	2	0.021	4	0.026
rs2165241														
Allele														
C	117	0.992	165	0.994	282	0.993	77	0.963	93	0.861	170	0.904	275	0.876
T	1	0.009	1	0.006	2	0.007	3	0.038	15	0.139	18	0.096	39	0.124
Genotype														
CC	58	0.983	82	0.988	140	0.986	37	0.925	40	0.741	77	0.819	122	0.777
CT	1	0.017	1	0.012	2	0.014	3	0.075	13	0.241	16	0.170	31	0.198
TT	0	0	0	0	0	0	0	0	1	0.019	1	0.011	4	0.026

EX, exfoliation syndrome; EG, exfoliation glaucoma; PG, primary open angle glaucoma aged 70 years or older; NG, normal tension glaucoma aged 70 years or older; and CT, cataract aged 70 years or older.

**Table 3:** Odds ratios and p values for allelic and genotypic frequencies of rs1048661, rs3825942, and rs2165241 in comparison between cases (EX, EG, and EX+EG) and controls (CT, PG, NG, and PG+NG).

**Parameter**	**EX**				**EG**				**EX+EG**			
	**Versus CT**	**Versus PG**	**Versus NG**	**Versus PG+NG**	**Versus CT**	**Versus PG**	**Versus NG**	**Versus PG+NG**	**Versus CT**	**Versus PG**	**Versus NG**	**Versus PG+NG**
rs1048661												
Allele												
p value	1.69x 10^–23^	3.23x 10^–13^	2.55x 10^–16^	4.08x 10^–19^	5.65x 10^–33^	1.92x 10^–17^	1.32x 10^–21^	4.71x 10^–26^	3.00x 10^–45^	2.61x 10^–20^	4.40x 10^–26^	1.18x 10^–33^
OR	19.71	16.67	18.39	17.64	28.23	23.88	26.35	25.27	23.97	20.27	22.37	21.45
95% CI	8.90–43.67	6.91–40.22	7.84–43.14	7.80–39.88	12.83–62.14	9.96–57.27	11.31–61.41	11.25–56.76	13.40–42.87	10.14–40.56	11.60–43.16	11.67–39.43
Genotype												
p value	4.11x 10^–25^	1.16x 10^–11^	1.91x 10^–17^	2.31x 10^–19^	4.80x 10^–32^	2.12x 10^–14^	6.50x 10^–21^	3.42x 10^–24^	3.78x 10^–43^	1.04x 10^–16^	2.74x 10^–25^	3.57x 10^–31^
rs3825942												
Allele												
p value	1.77x 10^–8^	2.10x 10^–33^	2.10x 10^–33^	2.10x 10^–33^	2.42x 10^–22^	6.48x 10^–8^	4.72x 10^–9^	1.23x 10^–10^	3.98x 10^–16^	8.68x 10^–10^	1.47x 10^–11^	1.84x 10^–14^
OR	28.21	29.25	29.93	29.64	39.78	41.25	42.21	41.8	34	35.25	36.07	35.72
95% CI	3.86–205.97	3.79–225.66	3.96–226.36	4.01–219.07	5.46–289–78	5.36–317.49	5.59–318.47	5.67–308.20	8.23–140.45	7.91–157.16	8.31–156.49	8.50–150.11
Genotype												
p value	2.10x 10^–33^	2.10x 10^–33^	2.10x 10^–33^	2.10x 10^–33^	3.53x 10^–11^	7.19x 10^–8^	2.88x 10^–9^	1.11x 10^–10^	6.37x 10^–16^	1.20x 10^–9^	1.00x 10^–11^	1.17x 10^–14^
rs2165241												
Allele												
p value	4.79x 10^–5^	0.3051	1.03x 10^–33^	1.20x 10^–3^	7.87x 10^–7^	0.1022	4.87x 10^–6^	8.28x 10^–5^	1.08x 10^–9^	0.0729	1.52x 10^–7^	3.02x 10^–6^
OR	16.59	4.56	18.87	12.39	23.4	6.43	26.61	17.47	20	5.49	22.74	14.93
95% CI	2.25–122.20	0.47–44.63	2.45–145.49	1.63–94.08	3.18–171.92	0.66–62.81	3.46–204.70	2.31–132.36	4.78–83.62	0.90–33.46	5.11–101.30	3.42–65.14
Genotype												
p value	1.95x 10^–4^	0.3	1.59x 10^–4^	2.82x 10^–3^	5.14x 10^–6^	0.1004	7.16x 10^–6^	1.23x 10^–4^	1.07x 10^–8^	0.0715	2.79x 10^–7^	4.71x 10^–6^

OR, odds ratio; CI, confidence interval; NA, not applicable; EX, exfoliation syndrome; EG, exfoliation glaucoma; PG, primary open-angle glaucoma aged 70 years or older; NG, normal tension glaucoma aged 70 years or older; and CT, cataract aged 70 years or older. p values were obtained by Fisher's exact probability test.

None of the three SNPs showed a significant difference between the EX and EG groups in these allelic or genotypic frequencies (Table 4). In addition to this, none of the three SNPs showed significant differences in their allelic or genotypic frequencies in comparisons between the primary glaucoma (PG, NG, or PG+NG) and CT groups or between the PG and NG groups (data not shown), excepted for the allele C of rs2165241 (p=0.0233) and the genotype CC of rs2165241 (p=0.0294) in comparison between the PG and NG groups.

**Table 4:** Odds ratios and p values for three SNPs in comparison between EX and EG.

	**EX versus EG**		
rs1048661	rs3825942	rs2165241
Allele			
p value	0.5829	1	1
OR	1.43	1.41	1.41
95% CI	0.49–4.20	0.09–22.78	0.09–22.78
Genotype			
p value	0.2005	1	1

No significant association was found between EX and EG. OR, odds ratio; CI, confidence interval; EX, exfoliation syndrome; and EG, exfoliation glaucoma. p values were obtained by Fisher's exact probability test.

The two SNPs, rs1048661 and rs3825942, were in linkage disequilibrium (D’=1). In our study populations, only three of the four possible haplotypes in rs1048661/rs3825942 were detected (Table 5 and Table 6). In the comparisons between cases (EX, EG, or EX+EG) and controls (CT+PG+NG), the T and G were significantly associated with EX, EG, and EX+EG, and the G and A had a significant protective effect (Table 5 and Table 6).

**Table 5:** Counts and frequencies of haplotype rs1048661/rs3825942 in cases (EX, EG, and EX+EG) and controls (CT, NG, PG+NG, CT+NG, and CT+PG+NG).

	**Cases**									
**EX**		**EG**		**EX+EG**					
**Count**	**Frequency**	**Count**	**Frequency**	**Count**	**Frequency**				
TG	111	0.941	159	0.958	270	0.951				
GG	6	0.051	6	0.036	12	0.042				
GA	1	0.008	1	0.006	2	0.007				
	**Controls**									
	**CT**		**NG**		**PG+NG**		**CT+NG**		**CT+PG+NG**	
	**Count**	**Frequency**	**Count**	**Frequency**	**Count**	**Frequency**	**Count**	**Frequency**	**Count**	**Frequency**
TG	140	0.443	50	0.463	89	0.473	190	0.448	229	0.454
GG	115	0.364	36	0.333	61	0.324	151	0.356	176	0.349
GA	61	0.193	22	0.204	38	0.202	83	0.196	99	0.196

EX, exfoliation syndrome; EG, exfoliation glaucoma; PG, primary open angle glaucoma aged 70 years or older; NG, normal tension glaucoma aged 70 years or older; and CT, cataract aged 70 years or older.

**Table 6:** P values for haplotype rs1048661/rs3825942 in comparisons between cases (EX, EG, and EX+EG) and controls (CT, NG, PG+NG, CT+NG, and CT+PG+NG).

	**EX**				
**Versus** **CT**	**Versus NG**	**Versus** **PG+NG**	**Versus** **CT+NG**	**Versus** **CT+PG+NG**
TG	9.56x10^-21^	NA	9.56x10^-21^	6.19x10^-17^	1.27x10^-21^
GG	9.69x10^-11^	NA	9.69x10^-11^	1.76x10^-10^	1.43x10^-10^
GA	1.01x10^-6^	NA	1.01x10^-6^	5.63x10^-7^	5.63x10^-7^
	**EG**				
	**Versus** **CT**	**Versus** **NG**	**Versus** **PG+NG**	**Versus** **CT+NG**	**Versus** **CT+PG+NG**
TG	1.83x10^-28^	4.95x10^-21^	3.06x10^-23^	9.77x10^-30^	4.40x10^-20^
GG	3.12x10^-15^	2.51x10^-11^	4.82x10^-12^	2.60x10^-15^	3.70x10^-15^
GA	5.63x10^-9^	8.10x10^-9^	4.08x10^-9^	3.02x10^-9^	2.36x10^-9^
	**EX+EG**				
	**Versus** **CT**	**Versus** **NG**	**Versus** **PG+NG**	**Versus** **CT+NG**	**Versus** **CT+PG+NG**
TG	1.23x10^-40^	7.84x10^-29^	1.24x10^-32^	5.89x10^-43^	8.27x10^-44^
GG	5.93x10^-22^	4.01x10^-15^	1.03x10^-16^	2.39x10^-22^	2.83x10^-22^
GA	1.17x10^-13^	3.98x10^-13^	9.33x10^-14^	3.68x10^-14^	2.25x10^-14^

Significant association was found between cases and controls. EX, exfoliation syndrome; EG, exfoliation glaucoma; PG, primary open angle glaucoma aged 70 years or older; NG, normal tension glaucoma aged 70 years or older; and CT, cataract aged 70 years or older. NA, not available due to a small number of samples.

## Discussion

**Figure 1:**
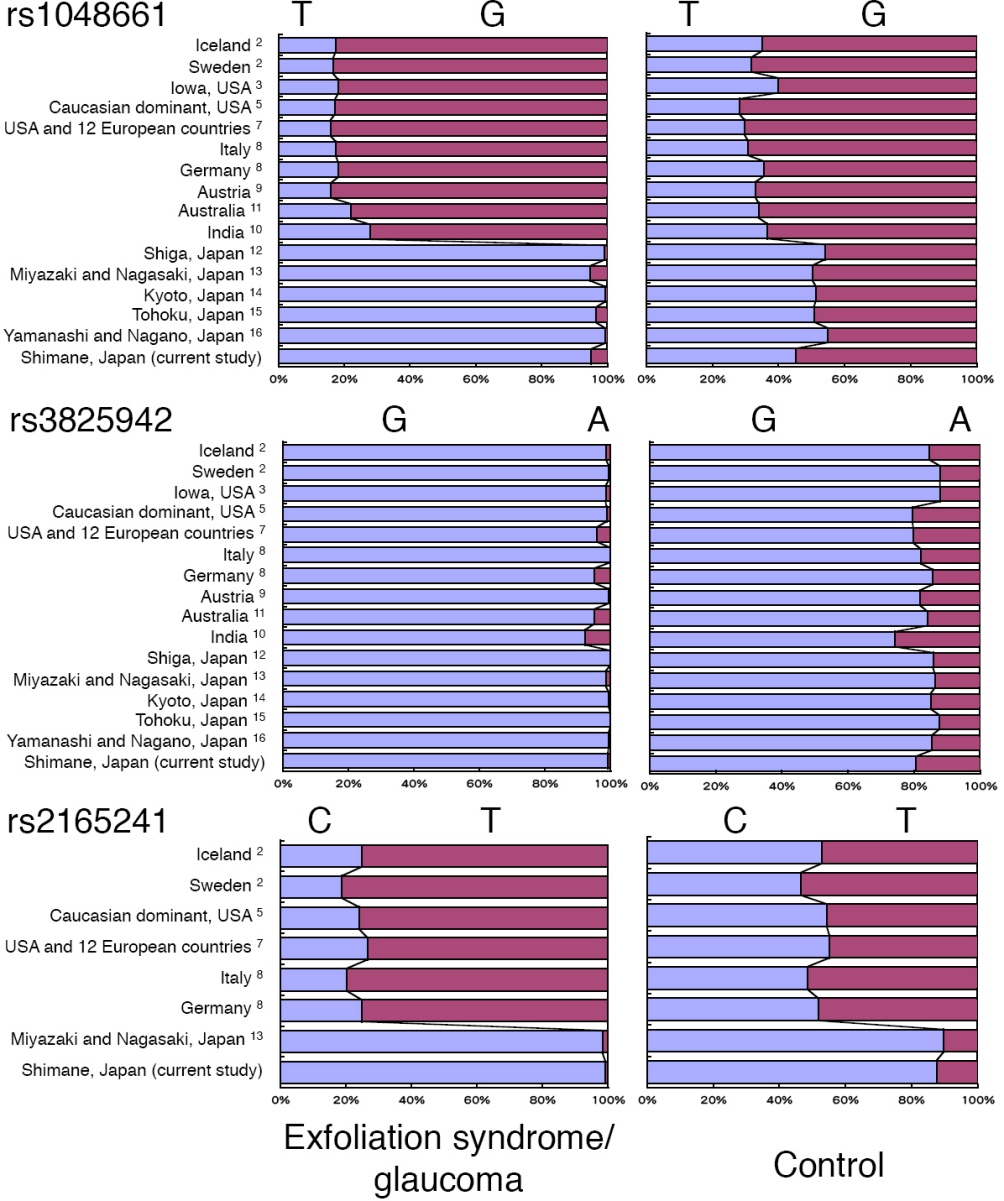
Allelic frequencies of the three SNPs reported in previous studies and the current study. Allelic frequencies of three SNPs in cases (exfoliation syndrome and exfoliation glaucoma) and controls (cataract or normal population) that have been reported in literatures are summarized.

Based on this study among 393 elderly Japanese patients with exfoliation syndrome, exfoliation glaucoma, primary open-angle glaucoma, and cataract, we confirmed the findings of Thorleifsson and colleagues [2] that three SNPs within *LOXL1* are strongly associated with exfoliation syndrome and glaucoma. In addition to the original study in Icelandic and Swedish populations, allele G of rs3825942 has been consistently suggested as a risk–associated allele of exfoliation syndrome/glaucoma in five studies from the United States [3-7], two studies from Europe [8,9], one study from India [10], one study from Australia [11], and six studies including this one from Japan [12-16]. In this study, we found that allele T of rs1048661 is associated with exfoliation syndrome/glaucoma, which is consistent with other studies in the Japanese population [12-16], while allele G is reported to be risk-associated in studies from other countries [2-4,6-11]. We also found that allele C of rs2165241 is associated with exfoliation syndrome/glaucoma, which is consistent with one study from Japan [13], while allele T is risk-associated in other populations [2,4,5,7,8]. Allelic frequencies of the three SNPs reported in previous studies and the current study are summarized in Figure 1. These results suggest a possibility that the missense changes in *LOXL1* are not actually causative but mark a haplotype that carries variants that may indeed be causative.

In this study, none of the SNPs exhibited significant differences in their allelic and genotypic frequencies between exfoliation syndrome and glaucoma, suggesting that these SNPs are associated with exfoliation glaucoma through their association with exfoliation syndrome as reported previously [2]. Accordingly, our data suggest that additional genetic or environmental risk factors are associated with the development of exfoliation glaucoma among exfoliation syndrome patients. Further study is required to clarify these risks.

This study revealed extremely high ORs and significant p values for the three SNPs in the comparisons between the exfoliation syndrome/glaucoma groups and cataract groups. Only patients aged 70 years or older were recruited as control subjects in this study, enabling us to reduce the chance of misclassifying latent or preclinical exfoliation syndromes into the control group. Accordingly, the inclusion criteria of the control group might contribute to the extremely significant association of the *LOXL1* SNPs with exfoliation syndrome in this study. In contrast, the significance of the association of the SNPs with exfoliation syndrome/glaucoma was relatively low when the PG group was used as a control comparison compared to when the NG or CT groups were used as control comparisons. Although the level of significance was relatively low, we found differences in frequencies between PG and NG for allele C of rs2165241 (p=0.0233) and the genotype CC of rs2165241 (p=0.0294). These may suggest the possible inclusion of late onset exfoliation syndrome in the PG group. Previously, a lack of association between *LOXL1* polymorphisms and primary open-angle glaucoma or primary angle-closure glaucoma were reported in Caucasian, African American, Ghanaian, and Indian populations [17,18]. We did not find any significant associations between any of the three SNPs with primary open-angle glaucoma, reconfirming the previous observations in our Japanese population. Most recently, one study from Japan reported a lack of association between *LOXL1* polymorphisms and primary open-angle glaucoma in two of three SNPs (e.g., rs1048661 and rs3825942) [16]. In addition to this, the current study suggests a lack of association between rs2165241 and primary open-angle glaucoma as well as a lack of association between any of the three SNPs with normal tension glaucoma.

In summary, we have demonstrated significant associations of *LOXL1* variants with Japanese patients who have exfoliation syndrome/glaucoma. Compared to other populations, the risk alleles in rs1048661 and rs2165241 are unique in this population. The *LOXL1* association of exfoliation glaucoma is through the association of exfoliation syndrome. *LOXL1* lacked any association with primary open-angle glaucoma or normal tension glaucoma in this population. Additional genetic or environmental risk factors other than *LOXL1* are likely to be associated with an increase in exfoliation glaucoma among exfoliation syndrome patients.

## References

[1] Ritch R, Schlotzer-Schrehardt U (2001). Exfoliation syndrome.. Surv Ophthalmol.

[2] Thorleifsson G, Magnusson KP, Sulem P, Walters GB, Gudbjartsson DF, Stefansson H, Jonsson T, Jonasdottir A, Stefansdottir G, Masson G (2007). Common sequence variants in the LOXL1 gene confer susceptibility to exfoliation glaucoma.. Science.

[3] Fingert JH, Alward WL, Kwon YH, Wang K, Streb LM, Sheffield VC, Stone EM (2007). LOXL1 mutations are associated with exfoliation syndrome in patients from the midwestern United States.. Am J Ophthalmol.

[4] Yang X, Zabriskie NA, Hau VS, Chen H, Tong Z, Gibbs D, Farhi P, Katz BJ, Luo L, Pearson E, Goldsmith J, Ma X, Kaminoh Y, Chen Y, Yu B, Zeng J, Zhang K, Yang Z (2008). Genetic association of LOXL1 gene variants and exfoliation glaucoma in a Utah cohort.. Cell Cycle.

[5] Fan BJ, Pasquale L, Grosskreutz CL, Rhee D, Chen T, DeAngelis MM, Kim I, del Bono E, Miller JW, Li T (2008). DNA sequence variants in the LOXL1 gene are associated with pseudoexfoliation glaucoma in a U.S. clinic-based population with broad ethnic diversity.. BMC Med Genet.

[6] Challa P, Schmidt S, Liu Y, Qin X, Vann RR, Gonzalez P, Allingham RR, Hauser MA (2008). Analysis of LOXL1 polymorphisms in a United States population with pseudoexfoliation glaucoma.. Mol Vis.

[7] Aragon-Martin JA, Ritch R, Liebmann J, O'Brien C, Blaaow K, Mercieca F, Spiteri A, Cobb CJ, Damji KF, Tarkkanen A (2008). Evaluation of LOXL1 gene polymorphisms in exfoliation syndrome and exfoliation glaucoma.. Mol Vis.

[8] Pasutto F, Krumbiegel M, Mardin CY, Paoli D, Lammer R, Weber BH, Kruse FE, Schlotzer-Schrehardt U, Reis A (2008). Association of LOXL1 Common Sequence Variants in German and Italian Patients with Pseudoexfoliation Syndrome and Pseudoexfoliation Glaucoma.. Invest Ophthalmol Vis Sci.

[9] Mossbock G, Renner W, Faschinger C, Schmut O, Wedrich A, Weger M (2008). Lysyl oxidase-like protein 1 (LOXL1) gene polymorphisms and exfoliation glaucoma in a Central European population.. Mol Vis.

[10] Ramprasad VL, George R, Soumittra N, Sharmila F, Vijaya L, Kumaramanickavel G (2008). Association of non-synonymous single nucleotide polymorphisms in the LOXL1 gene with pseudoexfoliation syndrome in India.. Mol Vis.

[11] Hewitt AW, Sharma S, Burdon KP, Wang JJ, Baird PN, Dimasi DP, Mackey DA, Mitchell P, Craig JE (2008). Ancestral LOXL1 variants are associated with pseudoexfoliation in Caucasian Australians but with markedly lower penetrance than in Nordic people.. Hum Mol Genet.

[12] Hayashi H, Gotoh N, Ueda Y, Nakanishi H, Yoshimura N (2008). Lysyl oxidase-like 1 polymorphisms and exfoliation syndrome in the Japanese population.. Am J Ophthalmol.

[13] Ozaki M, Lee KY, Vithana EN, Yong VH, Thalamuthu A, Mizoguchi T, Venkatraman A, Aung T (2008). Association of LOXL1 Gene Polymorphisms with Pseudoexfoliation in the Japanese.. Invest Ophthalmol Vis Sci.

[14] Mori K, Imai K, Matsuda A, Ikeda Y, Naruse S, Hitora-Takeshita H, Nakano M, Taniguchi T, Omi N, Tashiro K (2008). LOXL1 genetic polymorphisms are associated with exfoliation glaucoma in the Japanese population.. Mol Vis.

[15] Fuse N, Miyazawa A, Nakazawa T, Mengkegale M, Otomo T, Nishida K (2008). Evaluation of LOXL1 polymorphisms in eyes with exfoliation glaucoma in Japanese.. Mol Vis.

[16] Mabuchi F, Sakurada Y, Kashiwagi K, Yamagata Z, Iijima H, Tsukahara S (2008). Lysyl oxidase-like 1 gene polymorphisms in Japanese patients with primary open angle glaucoma and exfoliation syndrome.. Mol Vis.

[17] Chakrabarti S, Rao KN, Kaur I, Parikh RS, Mandal AK, Chandrasekhar G, Thomas R (2008). The LOXL1 gene variations are not associated with primary open-angle and primary angle-closure glaucomas.. Invest Ophthalmol Vis Sci.

[18] Liu Y, Schmidt S, Qin X, Gibson JR, Hutchins K, Santiago-Turla C, Wiggs JL, Budenz DL, Akafo S, Challa P (2008). Lack of Association between LOXL1 variants and Primary Open-angle Glaucoma in Three Different Populations.. Invest Ophthalmol Vis Sci.

[19] Barrett JC, Fry B, Maller J, Daly MJ (2005). Haploview: analysis and visualization of LD and haplotype maps.. Bioinformatics.

